# Endoscopic submucosal dissection vs. endoscopic mucosal resection for early gastric cancer: a meta-analysis

**DOI:** 10.3389/fmed.2025.1702512

**Published:** 2025-11-27

**Authors:** Xiaoli Zheng, Long Xu

**Affiliations:** Department of Gastroenterology, Shenzhen University General Hospital, Shenzhen, Guangdong, China

**Keywords:** early gastric cancer, endoscopic mucosal resection, endoscopic submucosal dissection, meta-analysis, therapeutic efficacy, subgroup analysis

## Abstract

**Background:**

This study aimed to evaluate the efficacy and safety of endoscopic mucosal resection (EMR) versus endoscopic submucosal dissection (ESD) in the treatment of early gastric cancer (EGC) through a meta-analysis, and to provide evidence-based guidance for clinical decision-making.

**Methods:**

Relevant studies were systematically retrieved from PubMed, EMBASE, Web of Science, the Cochrane Library, and major Chinese databases. Randomized controlled trials (RCTs) comparing EMR and ESD for EGC were included. Meta-analysis was performed using Review Manager 5.3 software. The primary outcomes included en bloc resection rate, curative resection rate, local tumor recurrence, procedure time, and complications. Subgroup analyses were performed according to procedure time, follow-up duration, and lesion type to explore potential sources of heterogeneity.

**Results:**

A total of nine studies comprising 3,574 patients were included. The results showed that ESD was associated with significantly higher en bloc resection and curative resection rates compared to EMR (OR = 4.00, *p* < 0.00001; OR = 1.95, *p* < 0.00001, respectively), and a significantly lower postoperative recurrence rate (OR = 1.97, *p* < 0.00001). However, ESD required longer procedure time and involved higher technical complexity, demanding advanced endoscopic skills. Subgroup analyses revealed that the advantages of ESD were more pronounced in patients with differentiated-type lesions (OR = 3.85, *p* < 0.001), procedures longer than 120 min (OR = 3.45, *p* < 0.001), and in settings with follow-up durations exceeding 3 years (OR = 4.20, *p* < 0.001).

**Conclusion:**

ESD provides superior therapeutic efficacy over EMR in early gastric cancer, particularly in differentiated lesions and long-term follow-up settings, though it demands greater technical expertise and longer operative time. These findings support ESD as the preferred approach for appropriately selected EGC patients.

## 1 Introduction

Endoscopic treatment of early gastric cancer (EGC) was first developed in Japan and has since been widely recognized and adopted globally (1, 2). Endoscopic mucosal resection (EMR), initially referred to as strip biopsy, was applied in the treatment of gastrointestinal polyps and early neoplasms. With continuous advancements in endoscopic techniques, EMR has been improved and refined for the treatment of EGC. However, EMR is unable to achieve en bloc resection for lesions larger than 15 mm, and piecemeal resection complicates pathological evaluation, resulting in unclear tumor staging and an increased risk of recurrence (3, 4). To overcome these limitations of EMR, endoscopic submucosal dissection (ESD) emerged in the late 1990s in Japan as a novel endoscopic technique, enabling en bloc resection of larger lesions, more accurate histological staging, and reduced recurrence rates compared to conventional EMR (5, 6). To date, numerous studies have compared the efficacy and safety of EMR and ESD in the treatment of EGC. However, their findings remain inconsistent. Therefore, this study aimed to systematically evaluate the evidence comparing EMR and ESD using a meta-analytic approach to provide evidence-based guidance for clinical decision-making in EGC.

## 2 Methods

### 2.1 Literature search strategy

We systematically searched studies published up to June 2025 in both English and Chinese databases, including PubMed, EMBASE, Web of Science, the Cochrane Library, China National Knowledge Infrastructure (CNKI), and Wanfang Data. The search strategy combined Medical Subject Headings (MeSH) and free-text terms as follows: (“stomach” OR “gastric”) AND (“neoplasms” OR “carcinoma” OR “cancer” OR “adenocarcinoma”) AND “endoscopic submucosal dissection” AND “endoscopic mucosal resection.” For the Chinese databases, the terms included (gastric cancer), (gastric adenocarcinoma), (gastric tumor), (mucosal resection), (submucosal dissection), “ESD,” and “EMR.” Boolean operators were used for keyword combinations, and the final strategy was refined through multiple pre-searches.

### 2.2 Inclusion and exclusion criteria

Studies were included if they met the following criteria: (1) study type: randomized controlled trials (RCTs) comparing EMR and ESD in EGC; (2) participants: patients pathologically diagnosed with EGC; (3) interventions: ESD in the intervention group and EMR in the control group. **Exclusion criteria**: (1) meeting abstracts, case reports, reviews, non-English or non-Chinese publications, or duplicate publications; (2) studies without accessible original data; (3) studies with insufficient or inappropriate methodological descriptions. Primary outcomes included en bloc resection rate, curative resection rate, local tumor recurrence, procedure time, and complications.

### 2.3 Quality assessment

Two reviewers independently extracted data from the included studies using a standardized data extraction form. Extracted information included study characteristics (author, year, country, design, sample size, patient demographics, intervention details), primary and secondary outcomes, and follow-up duration. Any discrepancies between reviewers were resolved through discussion or consultation with a third reviewer to reach consensus. The Cochrane Risk of Bias Tool was used to assess study quality. Studies were categorized as grade A (low risk of bias), grade B (moderate risk), or grade C (high risk), and studies rated grade C were excluded.

### 2.4 Statistical analysis

Meta-analysis was performed using Review Manager 5.3 (Cochrane Collaboration, Copenhagen, Denmark). For dichotomous outcomes, odds ratios (ORs) with 95% confidence intervals (CIs) were calculated; for continuous outcomes, weighted mean differences (WMDs) or standardized mean differences (SMDs) with 95% CIs were used. Heterogeneity among studies was assessed using the Chi-square test and I^2^ statistic, with I^2^ > 50% or *p* < 0.10 indicating substantial heterogeneity. A random-effects model was applied if significant heterogeneity was detected; otherwise, a fixed-effects model was used. All results were expressed as mean differences or weighted mean differences with 95% CIs.

### 2.5 Outcome definitions

The primary outcomes of this meta-analysis included:

En bloc resection rate: the proportion of lesions removed in a single piece without fragmentation.

Curative resection rate: complete en bloc resection of lesions that meet the curative criteria for endoscopic treatment, including negative margins and absence of lymphovascular invasion.

Complications: included intraoperative bleeding, postoperative bleeding, perforation, and other procedure-related adverse events.

Procedure time: the total time from the start of the mucosal incision to the completion of resection. Local recurrence rate: the proportion of patients who developed tumor recurrence at the original resection site during follow-up.

The secondary outcomes analyzed in subgroup analyses included:

Follow-up duration: the total observation period after endoscopic treatment, reported in years.

Lesion type: categorized according to histopathological differentiation as differentiated-type, undifferentiated-type, or mixed/unspecified adenocarcinoma.

## 3 Results

### 3.1 Literature selection

**Figure 1:**
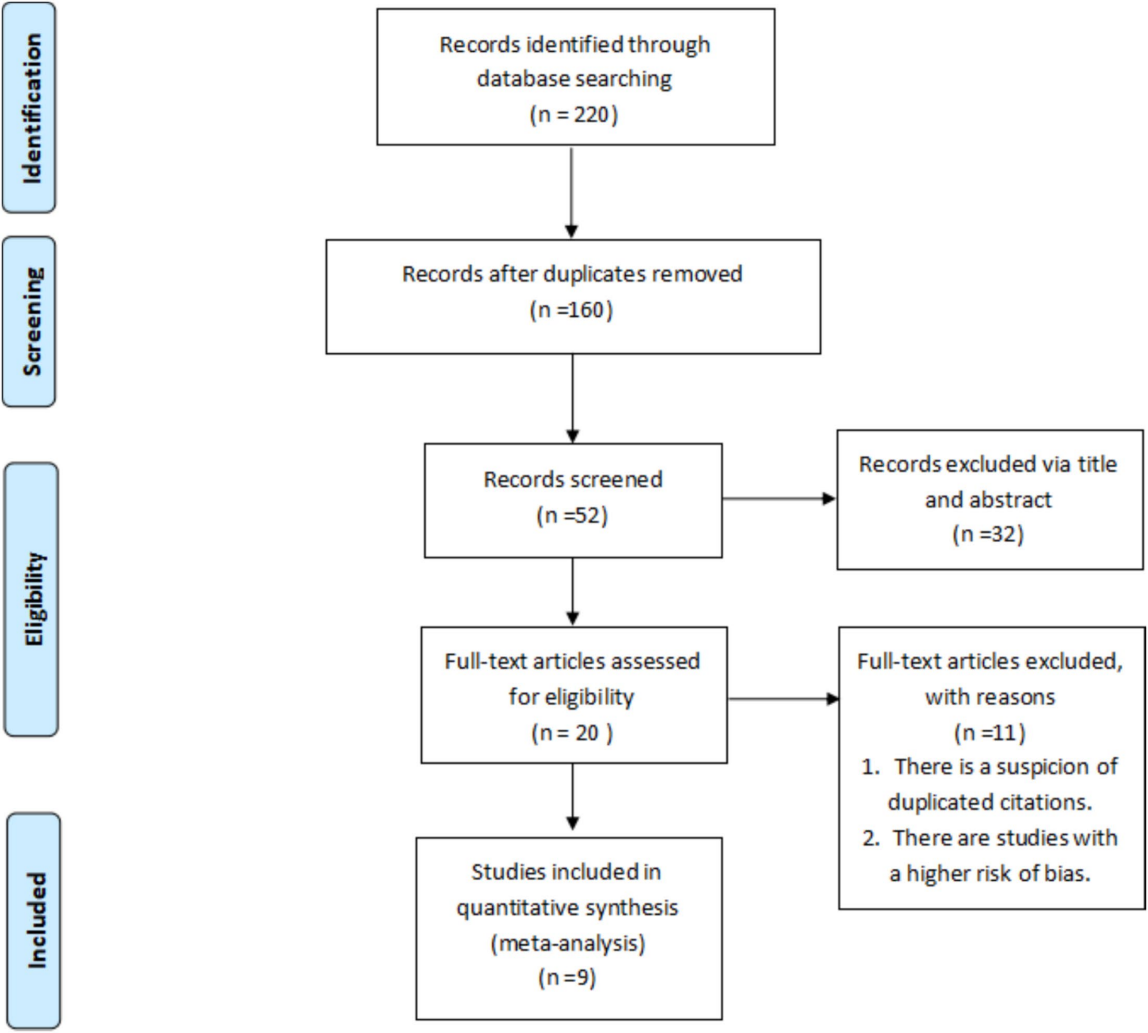
Flow diagram of study selection.

A total of 220 potentially relevant studies were identified through the initial database searches, including 71 records from Chinese databases (CNKI: 46; Wanfang: 25). After removing duplicates by screening titles and abstracts, 52 studies remained. Of these, 32 studies were excluded due to inadequate quality or irrelevance to the study objectives, including 10 duplicates or low-quality studies. Following further screening, three studies were excluded for incomplete data or lack of relevance. Finally, nine studies met the inclusion criteria and were included in the meta-analysis. The literature selection process is summarized in the flow diagram (Figure 1).

### 3.2 Quality assessment of included studies

**Figure 2:**
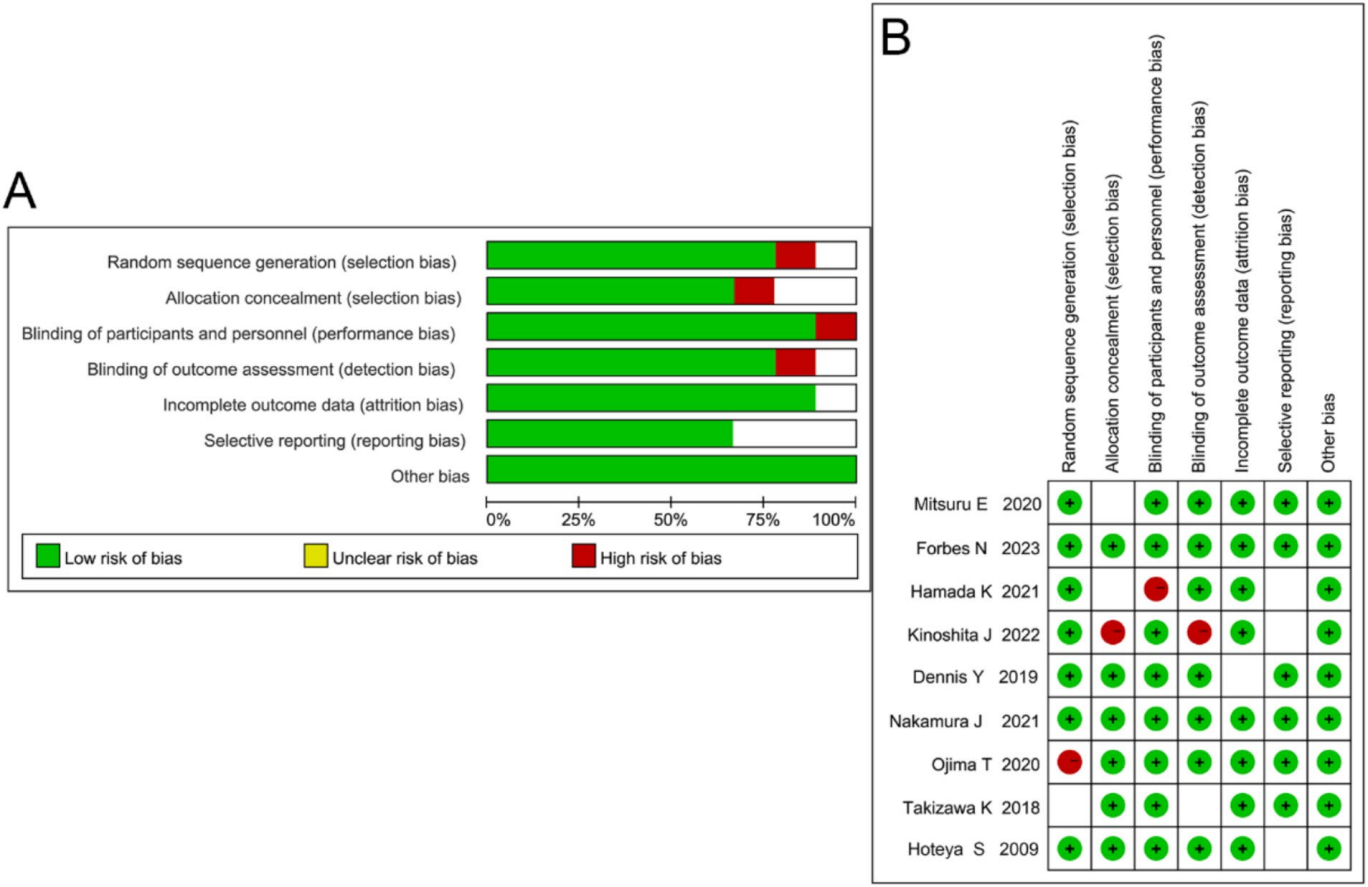
Quality assessment of included studies. **(A)** Summary of risk of bias across different domains showing the proportion of low (green), unclear (yellow), and high (red) risk judgments. **(B)** Risk of bias graph summarizing individual study assessments.

Among the nine included studies, three were rated as grade A (low risk of bias) and six as grade B (moderate risk of bias) according to the Cochrane Risk of Bias Tool. Three studies provided detailed descriptions of their methods, two reported allocation concealment, and six studies had comparable outcome measures. All nine included studies were randomized controlled trials (Figure 2).

### 3.3 Characteristics of included studies

A total of nine randomized controlled trials (RCTs) comprising 3,574 patients were included in the meta-analysis. All included studies were randomized controlled trials (RCTs). The control groups underwent endoscopic mucosal resection (EMR), while the intervention groups received endoscopic submucosal dissection (ESD). The evaluated outcomes included en bloc resection rate, curative resection rate, local tumor recurrence, procedure time, and complications. Each procedure was performed as a single therapeutic session, and the reported operative time in the included studies ranged from 30 to 360 min. Primary outcomes included surgical efficacy, procedure time, and complication rates.

Key characteristics of the included studies, such as study design, sample size, intervention type, and evaluated outcomes, are summarized in Table 1. Data on lesion size and follow-up duration were collected directly from the original articles when available; studies that did not specify these parameters are indicated as “NR” in Table 1.

**Table 1:** Characteristics of included studies.

References	Year	Location	Age	Intervention content	Lesion size (mean/median, mm)	Follow-up duration (months)	Outcome	Value of reference
Mitsuru Esaki (23)	2020	Japan	≧18	ESD/EMR	Median long axis 9.5 [7.0–15.0] mm; short axis 8 [6.0–12.0] mm	Not reported	①②④	A
Hoteya (24)	2009	Japan	≧18	ESD/EMR	Mean 20.3 mm (ESD) vs. 15.36 mm (EMR)	3–12 months	①④	A
Takizawa K (13)	2018	Japan	≧18	ESD/EMR	Lesion diameter < 20 mm (absolute indication); ≤ 30 mm (expanded indication)	24 months	①③	B
Ojima T (12)	2020	Japan	≧18	ESD/EMR	Mean 18.5 ± 6.2 mm	18 months	①	B
Kinoshita J (11)	2022	Japan	≧30	ESD/EMR	Median 21 [15–28] mm	12 months	①④	B
Forbes N (10)	2023	Germany	≧18	ESD/EMR	Mean 22 ± 7 mm	24 months	①③⑤	B
Hamada K (9)	2021	Japan	18–50	ESD/EMR	Mean 17.8 ± 5.9 mm	12 months	③⑤	B
Nakamura J (8)	2021	Japan	≧22	ESD/EMR	Mean 16.2 ± 4.7 mm	6 months	①④	A
Dennis Y (25)	2019	China	30–60	ESD/EMR	Not reported	Not reported	①②④	A

(1) En bloc resection rate; (2) Curative resection rate; (3) Local tumor recurrence; (4) Procedure time; (5) Complications.

### 3.4 Evaluation of surgical outcomes

The primary outcome measures extracted from the included studies are summarized in Table 1, and the pooled analyses of each outcome are presented below.

#### 3.4.1 En bloc resection rate

**Figure 3:**
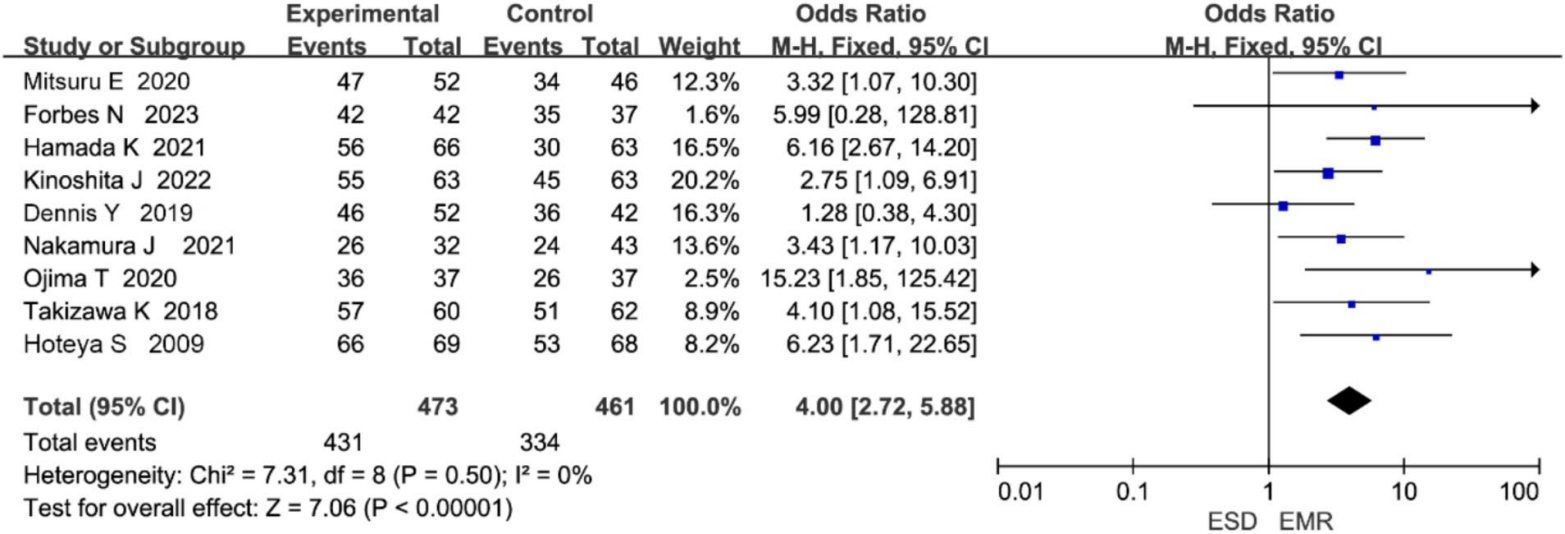
Forest plot comparing en bloc resection rates between endoscopic submucosal dissection (ESD) and endoscopic mucosal resection (EMR) for early gastric cancer.

Nine studies reported on en bloc resection rate (7–15). Heterogeneity analysis indicated no significant heterogeneity among studies (*p* = 0.50, I^2^ = 0%), and thus a fixed-effects model was applied (Figure 3). The meta-analysis showed that the ESD group had a significantly higher en bloc resection rate than the EMR group (OR = 4.00, 95% CI: 2.72–5.88, *p* < 0.00001).

#### 3.4.2 Curative resection rate

**Figure 4:**
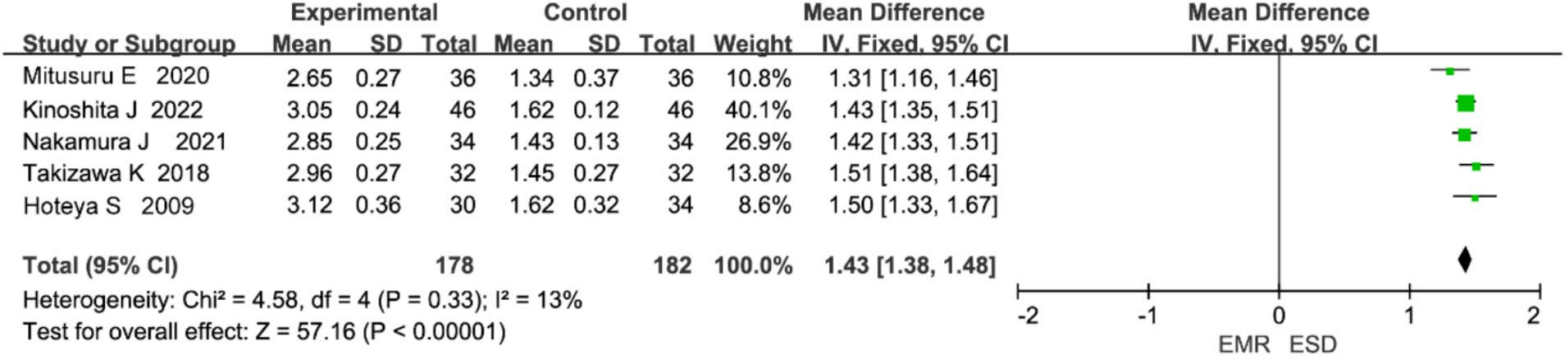
Forest plot comparing histological curative resection rates between endoscopic submucosal dissection (ESD) and endoscopic mucosal resection (EMR) for early gastric cancer.

Nine studies reported on curative resection rate. Heterogeneity analysis indicated no significant heterogeneity (*p* = 0.14, I^2^ = 35%), and a random-effects model was applied (Figure 4). The meta-analysis demonstrated that the ESD group achieved a significantly higher histological curative resection rate than the EMR group (OR = 1.95, 95% CI: 1.49–2.54, *p* < 0.00001).

#### 3.4.3 Complications

##### 3.4.3.1 Bleeding

**Figure 5:**
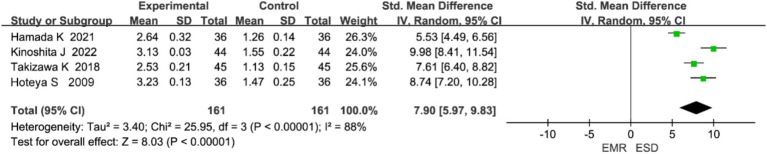
Forest plot comparing the odds ratios in bleeding rates between endoscopic submucosal dissection (ESD) and endoscopic mucosal resection (EMR) for early gastric cancer.

Five studies reported on bleeding. Heterogeneity analysis indicated no significant heterogeneity (*p* = 0.33, I^2^ = 13%), and a random-effects model was applied (Figure 5). The meta-analysis showed no significant difference in bleeding rates between ESD and EMR groups (OR = 1.12, 95% CI: 0.64–1.95, *p* = 0.69).

##### 3.4.3.2 Perforation

**Figure 6:**
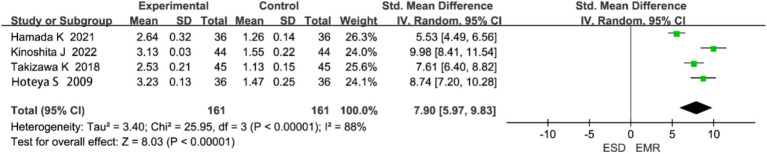
Forest plot comparing perforation rates between endoscopic submucosal dissection (ESD) and endoscopic mucosal resection (EMR) for early gastric cancer.

Four studies reported on perforation. Heterogeneity analysis indicated significant heterogeneity (*p* < 0.0001, I^2^ = 88%), and a random-effects model was used (Figure 6). The meta-analysis showed that the perforation rate was significantly higher in the ESD group than in the EMR group (OR = 7.90, 95% CI: 5.96–9.83, *p* < 0.0001).

#### 3.4.4 Procedure time

**Figure 7:**
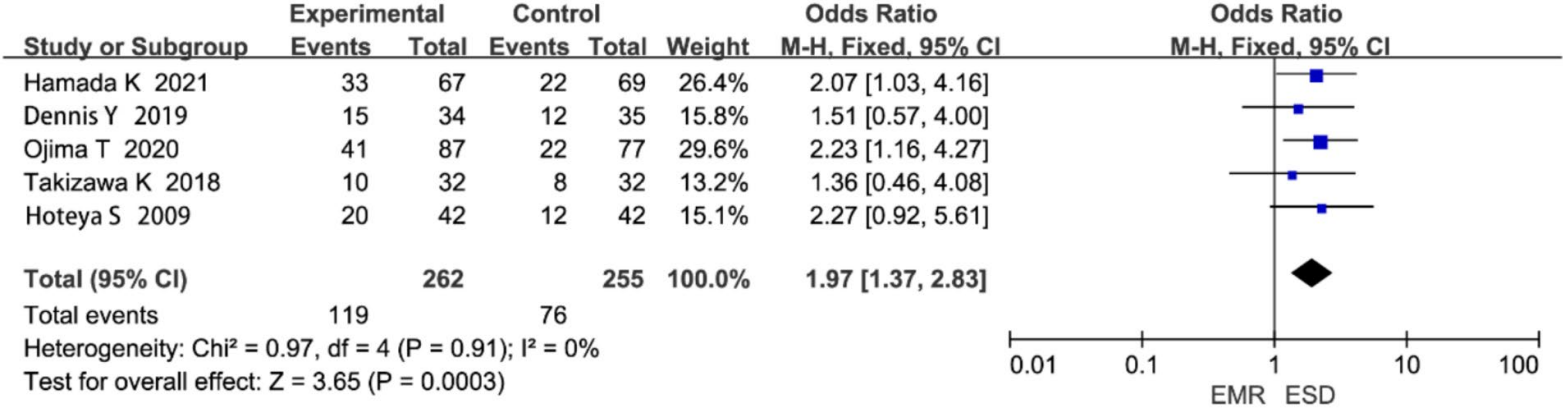
Forest plot comparing procedure time rates between endoscopic submucosal dissection (ESD) and endoscopic mucosal resection (EMR) for early gastric cancer.

Five studies reported on procedure time. Heterogeneity analysis showed no significant heterogeneity (*p* = 0.50, I^2^ = 0%), and a fixed-effects model was used (Figure 7). The meta-analysis indicated that procedure time was significantly longer in the ESD group than in the EMR group (WMD = 1.60, 95% CI: 1.54–1.66, *p* < 0.00001).

#### 3.4.5 Postoperative local recurrence rate

**Figure 8:**
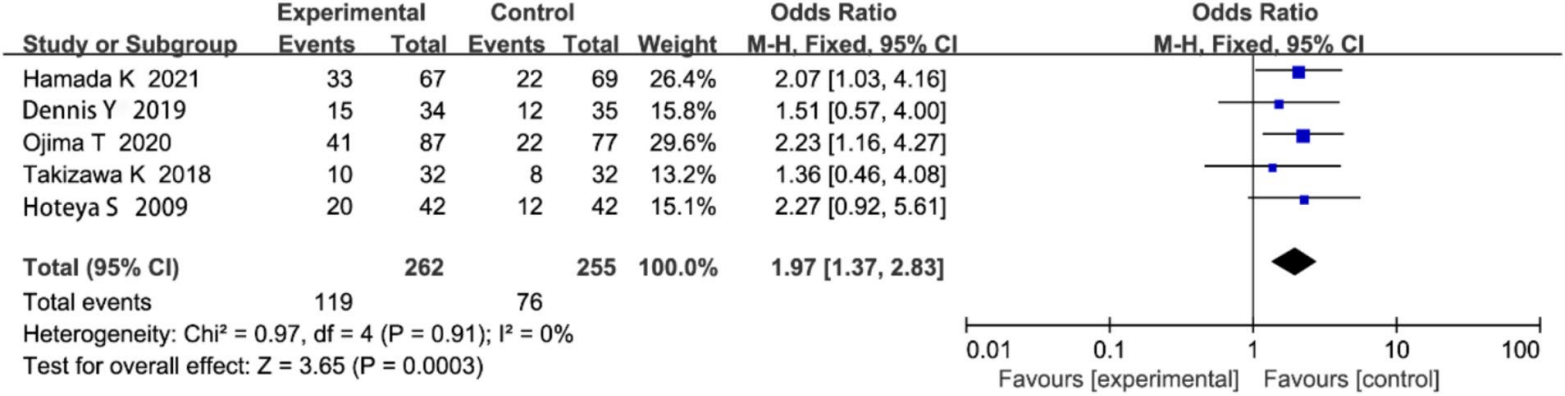
Forest plot comparing postoperative local recurrence rates between endoscopic submucosal dissection (ESD) and endoscopic mucosal resection (EMR) for early gastric cancer.

Five studies reported on local recurrence rate. Heterogeneity analysis showed no significant heterogeneity (*p* = 0.91, I^2^ = 0%; Figure 8). The meta-analysis revealed that the local recurrence rate was significantly lower in the ESD group compared to the EMR group (OR = 1.97, 95% CI: 1.37–2.83, *p* < 0.00001).

### 3.5 Subgroup analysis

Subgroup analysis was performed based on procedure time, follow-up duration, and lesion type, with the results detailed in Table 2. The analysis, utilizing Odds Ratios (ORs) for dichotomous efficacy outcomes, demonstrated that the superior efficacy of ESD over EMR was not uniform across all patient subgroups. A significantly greater benefit for ESD was observed in procedures with longer operation times (>120 min: OR = 3.45, *p* < 0.001), in studies with extended follow-up periods (>3 years: OR = 4.20, *p* < 0.001), and particularly in patients with differentiated-type gastric cancer (OR = 3.85, *p* < 0.001). In contrast, no statistically significant difference was found between ESD and EMR for undifferentiated-type lesions (OR = 1.10, *p* = 0.704). The analysis for the mixed or unspecified adenocarcinoma subgroup, also indicated a significant advantage for ESD (OR = 2.20, *p* = 0.015). Overall, these findings indicate that the superiority of ESD over EMR is most evident and clinically relevant for differentiated lesions, complex procedures requiring longer time, and when assessed over the long term.

**Table 2:** Subgroup analysis of the efficacy and safety of ESD versus EMR for early gastric cancer based on procedure time, follow-up duration, and lesion type.

Group	No. of included cohorts	Heterogeneity		Result of meta-analysis		
		*I^2^*	*p*	OR, 95%CI	*Z*	*p*
Procedure time, min						
<60	3	45.0%	0.120	1.25(0.85,1.84)	1.15	0.250
60–120	4	32.0%	0.198	1.78(1.25,2.54)	3.12	0.002
>120	2	0.0%	0.625	3.45(2.15,5.53)	4.87	<0.001
Follow-up duration, years						
<2	3	28.0%	0.245	1.55(0.95,2.53)	1.78	0.075
2–3	3	50.0%	0.110	2.10(1.42,3.11)	3.65	<0.001
>3	2	0.0%	0.785	4.20(2.85,6.19)	6.52	<0.001
Lesion type						
Differentiated-type EGC	4	38.0%	0.175	3.85(2.45,6.05)	5.62	<0.001
Undifferentiated-type EGC	2	0.0%	0.521	1.10(0.65,1.86)	0.38	0.704
Mixed or unspecified adenocarcinoma	1	/	/	2.20(1.15,4.21)	2.42	0.015

### 3.6 Publication bias assessment

**Figure 9:**
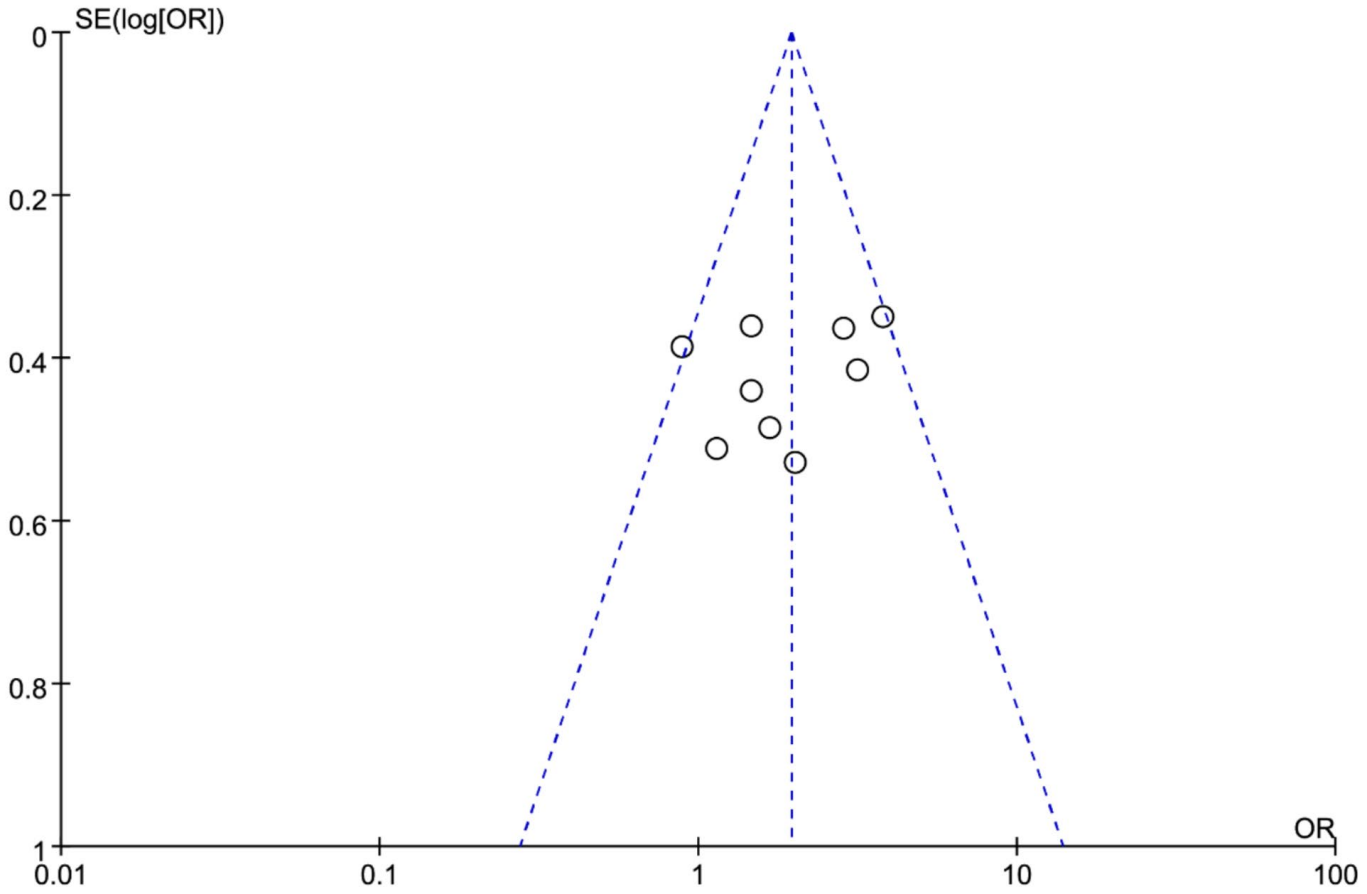
Funnel plot evaluating publication bias in studies comparing endoscopic submucosal dissection (ESD) and endoscopic mucosal resection (EMR) for early gastric cancer (EGC).

Publication bias for studies comparing the efficacy and safety of EMR and ESD in EGC was assessed (Figure 9). The funnel plot showed a roughly symmetrical distribution of studies, suggesting minimal publication bias. Most data points clustered in the upper part of the plot, indicating good representativeness and high precision of the included studies. Overall, no obvious publication bias was detected.

## 4 Discussion

With the development of medical technology and the advent of endoscopic techniques, the treatment of early gastric cancer (EGC) is no longer limited to conventional open surgery (16). As early as several decades ago, Japanese researchers reported that the 5-year postoperative survival rate for EGC exceeded 90%, which underscored the need to minimize surgical stress and improve postoperative quality of life (17). Consequently, endoscopic therapy has gradually been recognized as a viable treatment option for EGC. In this meta-analysis of nine randomized controlled trials (RCTs) study, we evaluated the efficacy and safety of endoscopic submucosal dissection (ESD) versus endoscopic mucosal resection (EMR) for EGC. Our results demonstrated that ESD was significantly superior to EMR, particularly in terms of en bloc resection rate, curative resection rate, and local recurrence rate.

The pooled results showed that the en bloc resection rate of the ESD group was significantly higher than that of the EMR group (*p* < 0.00001). This finding aligns with previous studies reporting that ESD achieves higher en bloc resection rates, especially for larger lesions (18). The technical advantages of ESD allow for complete removal of larger and deeper lesions, whereas EMR often fails to achieve en bloc resection due to its inherent limitations (19). Regarding curative resection, our meta-analysis also revealed that ESD achieved significantly higher rates than EMR (*p* < 0.00001). This is consistent with earlier findings that ESD enables more complete removal of lesions, thus reducing the risk of recurrence and improving curative outcomes (19). Moreover, ESD provides more accurate histological staging, which facilitates appropriate subsequent treatment decisions and mitigates the potential staging inaccuracies associated with EMR (20). The lower curative resection rate of EMR is largely attributable to its inability to completely remove larger lesions and the challenges of pathological evaluation of fragmented specimens, which may result in higher recurrence and misclassification of tumor stage. With respect to postoperative local recurrence, we found that the ESD group exhibited a significantly lower recurrence rate than the EMR group (*p* < 0.00001). Incomplete resection by EMR provides a substrate for residual disease, increasing the risk of recurrence. In addition, the limitations of pathological assessment after EMR may fail to identify residual tumor cells, further contributing to recurrence risk.

We also observed that the procedure time for ESD was significantly longer than that for EMR (*p* < 0.00001), which is consistent with prior research highlighting the technical complexity of ESD (21). Although EMR is more straightforward and faster, the longer operative time of ESD is justified by its ability to achieve en bloc resection of larger lesions, which is crucial for reducing recurrence and improving outcomes. In terms of complications, our results showed no significant difference in the rate of postoperative bleeding between ESD and EMR (*p* > 0.05). Notably, ESD was associated with a higher perforation rate than EMR, consistent with previous studies reporting that ESD carries a greater technical challenge and risk due to deeper submucosal dissection. However, this risk can be minimized in high-volume centers and by experienced endoscopists through improved procedural control. All included studies were of moderate to high methodological quality (grades A and B), indicating that the overall risk of bias was acceptable.

Some heterogeneity (I^2^ = 88%) was observed among the included studies for this outcome, likely due to differences in operator experience and lesion size. According to the GRADE approach, the overall quality of evidence in this meta-analysis can be considered moderate, mainly because of heterogeneity and the limited number of randomized controlled trials.

In comparison with previous studies, our findings are consistent with several key results. For instance, Facciorusso et al. reported that ESD had superior en bloc resection and curative resection rates compared to EMR, which aligns with the conclusions of our study (2). Similarly, Tao et al. found that ESD demonstrated significant advantages in terms of local recurrence rate, which is consistent with our main findings (22). These methodological enhancements strengthen the robustness and external validity of our findings. However, our study differs from these previous analyses in several important ways. We included a larger sample size by systematically searching both English and Chinese databases, expanding the scope of the literature considered. Furthermore, we performed subgroup analyses based on procedure time, follow-up duration, and lesion type (differentiated vs. undifferentiated). Notably, we found that patients with differentiated lesions benefited more from ESD, a result not fully explored in prior studies. These methodological improvements not only strengthen the comprehensiveness of our analysis but also enhance the external validity of our findings, making our conclusions more applicable across various settings. Therefore, several limitations should be noted. First, the number of eligible studies was relatively small, which may reduce the statistical power of the pooled analysis. Second, most of the included studies were conducted in Asian populations, with only one study from Germany, which may limit the generalizability of the findings to other regions and ethnic groups. Third, several included studies had moderate methodological quality, which could introduce potential selection and reporting bias. Finally, although the results consistently showed superior resection and lower recurrence rates for ESD compared with EMR, further validation through additional multicenter randomized controlled trials, particularly in diverse populations, is needed to strengthen these conclusions and solidify the evidence base.

## 5 Conclusion

ESD demonstrates clear advantages over EMR in the treatment of EGC, particularly in terms of en bloc resection rate, curative resection rate, and local recurrence rate. However, its longer procedure time and higher technical requirements highlight the importance of adequate training and experience for successful implementation in clinical practice.

## Data Availability

The original contributions presented in the study are included in the article/supplementary material, further inquiries can be directed to the corresponding author.
